# Bioinformatics-based analysis of potential candidates chromatin regulators for immune infiltration in osteoarthritis

**DOI:** 10.1186/s12891-022-06098-8

**Published:** 2022-12-23

**Authors:** Weiwei Wang, Zhixue Ou, Jianlan Peng, Ning Wang, Yi Zhou

**Affiliations:** 1 Guilin Hospital of Traditional Chinese Medicine, Guilin, 541002 Guangxi China; 2 Ruikang Hospital Affiliated to Guangxi University of Traditional Chinese Medicine, Nanning, 530001 Guangxi China; 3 The First Affiliated Hospital of Guangxi University of Traditional Chinese Medicine, Nanning, 530001 Guangxi China

**Keywords:** Chromatin regulators, Osteoarthritis, Immune infiltration, Bioinformatics, BRD1

## Abstract

**Background:**

Through the bioinformatics analysis to screen out the potential chromatin regulators (CRs) under the immune infiltration of osteoarthritis (OA), thus providing some theoretical support for future studies of epigenetic mechanisms under OA immune infiltration.

**Methods:**

By integrating CRs and the OA gene expression matrix, we performed weighted gene co-expression network analysis (WGCNA), differential analysis, and further screened Hub genes by protein-protein interaction (PPI) analysis. Using the OA gene expression matrix, immune infiltration extraction and quantification were performed to analyze the correlations and differences between immune infiltrating cells and their functions. By virtue of these Hub genes, Hub gene association analysis was completed and their upstream miRNAs were predicted by the FunRich software. Moreover, a risk model was established to analyze the risk probability of associated CRs in OA, and the confidence of the results was validated by the validation dataset.

**Results:**

This research acquired a total of 32 overlapping genes, and 10 Hub genes were further identified. The strongest positive correlation between dendritic cells and mast cells and the strongest negative correlation between parainflammation and Type I IFN reponse. In the OA group DCs, iDCs, macrophages, MCs, APC co-inhibition, and CCR were significantly increased, whereas B cells, NK cells, Th2 cells, TIL, and T cell co-stimulation were significantly decreased. The risk model results revealed that BRD1 might be an independent risk factor for OA, and the validation dataset results are consistent with it. 60 upstream miRNAs of OA-related CRs were predicted by the FunRich software.

**Conclusion:**

This study identified certain potential CRs and miRNAs that could regulate OA immunity, thus providing certain theoretical supports for future epigenetic mechanism studies on the immune infiltration of OA.

**Supplementary Information:**

The online version contains supplementary material available at 10.1186/s12891-022-06098-8.

## Background

Osteoarthritis (OA) is a common chronic clinical joint disease associated with synovial inflammation, bone fragmentation, abnormal subchondral bone remodeling, and articular cartilage degradation [1, 2]. According to studies, the prevalence of OA among adults in the USA can reach 10.4% [3]. It also affects approximately half of the world’s population over the age of 65 years and imposes a huge economic burden on our society [4, 5]. With the persistent development of modern medicine, the immune system has been discovered to be vital for the developmental process of OA. Immune cell infiltration mediates the autoimmune response in OA, inducing the secretion of chemokines, pro-inflammatory cytokines, and proteases, which in turn disrupt the immune homeostasis to accelerate cartilage erosion [6–8]. In addition, the onset and progression of OA are negatively affected by epigenetic dysregulation. Epigenetics is a mechanism that alters chromosomes, gene expression, and genetic phenotypes without altering DNA sequences [9], in which chromatin regulators (CRs) are essential upstream regulatory elements [10]. CRs can be separated into three primary categories on the basis of their roles in epigenetics: DNA methylators, histone modifiers, and chromatin remodelers [11].

These three types of CRs have been discovered to exert pivotal regulatory effects on the complex biological activity of osteoarthritis. The research finished by Xiong et al. [12] revealed that DNA methyltransferase 3β could inhibit miR-34a by promoting miR-34a methylation, which in turn effectively reduced cartilage extracellular matrix degradation and inflammatory responses in mice by targeting myeloid cell leukemia 1 and mediating the downstream *PI3K*/*AKT* pathway. *SIRT6* is a component of the Sirtuin6 family of proteins and is a class III histone deacetylase [13]. A previous human OA study unraveled that *SIRT6* was lowly expressed in articular cartilage, and further mouse experiments revealed that the overexpression of *SIRT6* in mice effectively reduced the expression of chemokines and pro-inflammatory cytokines, thereby decreasing cartilage damage [14]. Lysine-specific demethylase-1 (*LSD1*) is a chromatin-remodeling enzyme which regulates gene expression by demethylating *H3K9* [15]. Previously, researches have unveiled that high expression of *LSD1* leads to the demethylation of the microsomal prostaglandin E synthase 1 promoter *H3K9*, which induces the expression of OA chondrocyte-associated genes [16].

Although studies on the relevance of CRs in the field of OA have been conducted, few researches have systematically explored the relationship between CRs and OA, especially in the context of OA immune infiltration. Thereby, the present study firstly screened candidate key genes (overlapping genes) of CRs in OA via weighted gene co-expression network analysis (WGCNA) and differential analysis, and further screened Hub genes by protein-protein interaction (PPI) analysis. Based on the obtained Hub genes, the correlations between them and immune microenvironment were analyzed, and risk models were established as well. In addition, the current study also used the FunRich software to perform the miRNAs prediction of Hub genes, thus providing a theoretical basis for the mechanistic study on the epigenetics in OA.

## Method

### Genetic data sources and pre-processing

In order to obtain the gene microarray data related to OA, this study searched and screened the Gene Expression Omnibus (GEO) database (www.ncbi.nlm.nih.gov/geo/) using “Osteoarthritis” as the search term. The inclusive criteria were as follows: (1) mRNA expression profiles by array or high-throughput sequencing; (2) tissue samples in the dataset should be harvested from human subjects and include both normal controls and OA patient groups; (3) the total sample size of healthy and OA groups should be 70 cases or more. Based on the above criteria, we retrieved two qualified datasets from human peripheral blood samples, which include a training set GSE63359 based on the GPL96 platform and a validation set GSE48556 based on the GPL6947 platform. The GSE63359 dataset contains 72 samples, including 46 samples from OA patients and 26 samples from healthy individuals. The GSE48556 dataset contains 139 samples, including 106 samples of OA patients and 33 samples of healthy individuals. After we downloaded the raw and series matrix files of the corresponding datasets, the probe expression matrix was converted into a gene expression matrix using the xiantao academic platform (https://www.xiantao.love/). The chromosomal regulator genes were then derived from Lu et al. [10] By integrating the OA gene expression matrix and the CR file (Table. S1), the CR expression matrix was obtained.

### Differential expression analysis of CRs

In order to reveal the differences in the expressions of CRs between normal subjects and patients with osteoarthritis, we analyzed the CRs expression matrix of the training set using the “Limma” R language package. The fold change (FC) denotes the ratio of the expressing levels between these 2 groups, while log2FC represents the FC with a log base of 2, and the *P*-value is the threshold of significant difference. *P* < 0.05 and |log2FC| > 0.2 [17] were utilized as thresholds to screen differentially expressed genes. Meanwhile, the “pheatmap” and “ggplot2” R language packages were employed to create the heat map and volcano map, respectively, to visualize the differences.

### Gene co-expression analysis

Using the “WGCNA” package in R language, a co-expression network was established based on 46 OA patients and 26 healthy individuals via the following steps: (1) We defined the similar matrix; (2) We selected the weight coefficients β to transform the similar matrix into the adjacent matrix; (3) We transformed the adjacent matrix into a topological overlap matrix (TOM); (4) We performed hierarchic clustering on the Tom-based dissTOM to obtain a hierarchic cluster tree; (5) We identified the modules by the kinetic shear tree cutting approach (each co-expression module with at least 30 genes); (6) We computed the eigengene for every module (ME) and calculated the Pearson correlative coefficient between the MEs of every module. The co-expression network was obtained by combining the modules with high similarity. After the construction, we selected the core modules with the largest correlation coefficients between module eigenvectors and traits as well as the module with *P* < 0.05.

### Enrichment analysis of candidate key genes

According to the outcomes of differential genes and the outcomes of WGCNA core module genes, the Venn diagram was drawn through the “ggplot2” R language packages, and the intersection genes of the two were considered the candidate key genes in OA-related chromosomal regulators. Gene set enrichment analysis is an important analytical task for classifying common biological insights [18]. Here, we perform gene ontology as well as functional enrichment (biological processes, cellular components and molecular functions) and pathway enrichment studies of candidate key genes using the comprehensive and integrated gene set enrichment web tool DAVID (http://david.ncifcrf.gov) to understand the biological mechanisms and signaling pathways of candidate key genes [19–21]. Among them, the *P*-value < 0.05 was considered as a standard indicator to quantify the most important biological mechanisms and pathways. In addition, we also plotted enrichment analysis chord diagram with the “ggplot2” and “GOplot2” R language packages.

### Hub gene screening

To obtain the Hub genes in OA-related CRs, the candidate key genes were analyzed using the String Database (http://string-db.org/cgi/input.pl) with a confidence score > 0.4, and the outcomes were exported, input into Cytoscape 3.7.2 subsequently to establish PPI analysis maps. The cytoHubba plugin was employed to filter the key protein module, and the top 10 CRs with Score values were selected as Hub genes.

### Extraction and quantification of infiltrating immunocytes and functions

In order to extract and quantify the infiltrating immunocytes and biofunctions for the CRs expression matrix of the training set, we entered the keyword “IMMUNITY” in The Molecular Signatures Database (MsigDB) (https://www.gsea-msigdb.org/gsea/msigdb/) and searched for the corresponding gmt file. Then, the single-sample gene set enrichment analysis (ssGSEA) was completed on the CRs expression matrix using the “GSVA” R package. The ssGSEA is an immune infiltration analysis tool containing 29 immune scores to estimate the level of immunocyte infiltration in every specimen and it’s utilized to plot the immune infiltration percentage chart with the “reshape2”, “ggplot2”, and “RColorBrewer” packages in R language.

### Correlation and differential analysis between immune infiltration

To reveal the correlation between immunocytes and the correlation between immune functions, we performed correlation analysis on the basis of the ssGSEA expression matrix results with the “corrplo” packages in R language, with a heat map plotted eventually. In addition, to contrast the diversities in immunocytes and functions between the OA and control groups, we integrated the sample grouping and the ssGSEA expression matrix results, performing the rank sum test by the “vioplot” R language package and plotting violin plots eventually.

### Analysis of correlation between hub genes and immunocyte infiltration

In order to reveal the relationship of Hub genes in immunocyte infiltration, the “psych” R language package was utilized to study the result files of ssGSEA and Hub genes, and a correlation heat map was drawn. The top five Hub genes closely related to immune infiltration were selected as risk factors for subsequent analyses.

### Risk model construction and validation

To further analyze the results of the relationship between these five Hub genes and immunocyte infiltration, we used the CRs expression matrix and the sample grouping as input files to establish a logistic regression risk model and calculate the median value, which was higher than the median value and lower. Using the “rms” R language package, nomogram risk prediction plots were drawn to calculate the risk score for each of the five Hub genes, and they were validated by the calibration curve and receiver operating characteristic (ROC) curve to measure the identification ability of the nomogram model. The validation set GSE48556 is likewise used to construct logical risk models to assess the credibility of the analysis results. In addition, we also calculated the *K* values by the Kappa function to assess whether there was multicollinearity among the five genes. We consider that the *K* < 100 indicates the presence of a small degree of multicollinearity, 100 < *K* < 1000 indicates the presence of a moderate degree of multicollinearity; and when the *K* > 1000 indicates the presence of severe multicollinearity.

### Hub gene miRNA prediction

FunRich is a software for the analysis of protein gene function enrichment and interaction network. To further understand the expression mechanism of OA immune infiltration-related CRs, the FunRich software was utilized to predict their upstream miRNAs, and the miRNA-mRNA relationship was eventually input into the xiantao academic platform to visualize its regulatory network.

## Results

### Results of differential expression analysis of CRs

**Fig. 1:**
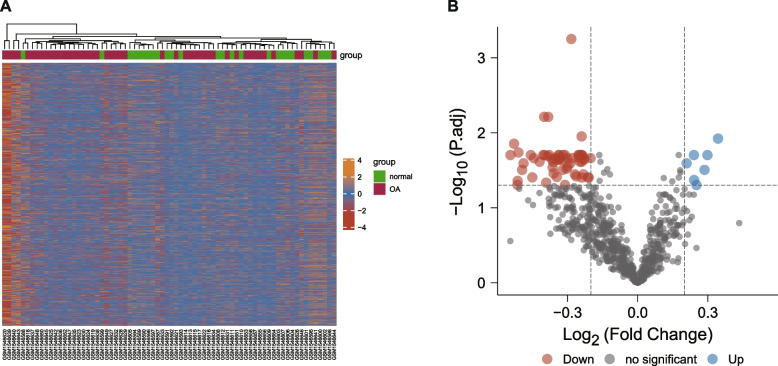
Diagram of differential gene expression analysis. **A **Heatmap of DEGs. The horizontal coordinates indicate the sample clusters. The more identical genetic expression levels between specimens, the closer they are in the graph. The color scale denotes the richness of genetic expression. The yellow color denotes upregulation and the red color indicates downregulation. **B **Volcanic map. The red dots indicate downregulated DEGs; the blue dots indicate upregulated DEGs; the gray dots indicate genes with no differential expression

Based on the CRs expression matrix of the training set, we screened the differentially expressed genes (DEGs) of CRs in OA and visualized the results by the heat map and volcano map (Fig. 1 A and 1 B). An overall 65 DEGs were identified in the present research, which included 7 upregulated and 58 downregulated genes (Table. S2).

### Gene co-expression analysis

**Fig. 2:**
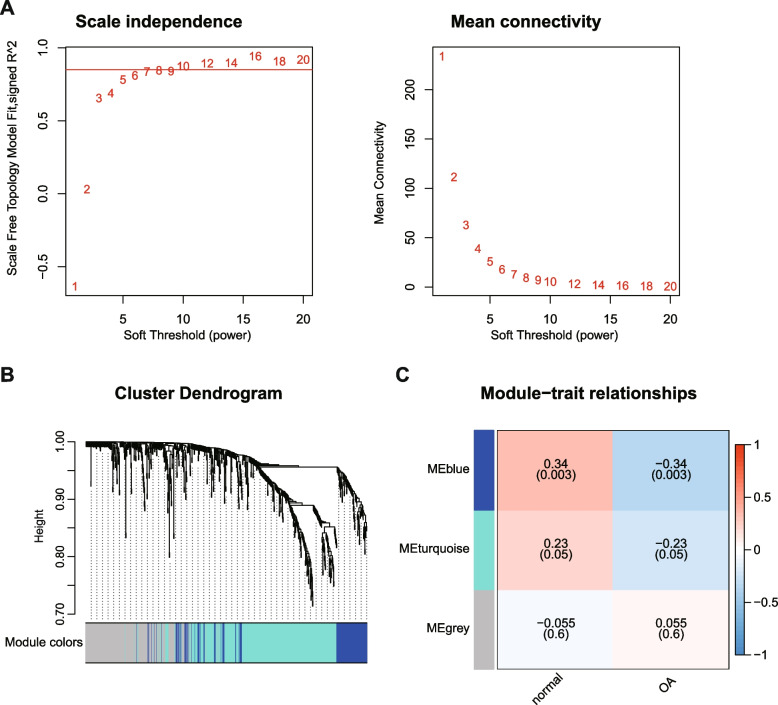
Gene coexpression analyses. **A **Soft threshold screening. **B **Hierarchic cluster plot. **C **Trait relationship plot

The WGCNA was completed by the “WGCNA” package of R language. First, for the sake of making the established net more coherent with the features of scale-free networks, we chose a soft threshold β = 10 (R^2^ = 0.85) for subsequent analysis (Fig. 2A). By establishing co-expression network modules, an overall 3 coexpression modules were obtained. There were at least 30 genes in each module. The outcomes were demonstrated in a hierarchic cluster plot (Fig. 2B). A total of 217 of these genes weren’t grouped into any module and were denoted in gray (Table. S3). Moreover, through investigating the trait relationship between modules and OA, the most significantly correlated module Blue (R = − 0.34, *P* = 0.003) was extracted as the key module, which contained a total of 132 genes (Fig. 2C, and Table. S3).

### Results of candidate key gene enrichment analysis

**Fig. 3:**
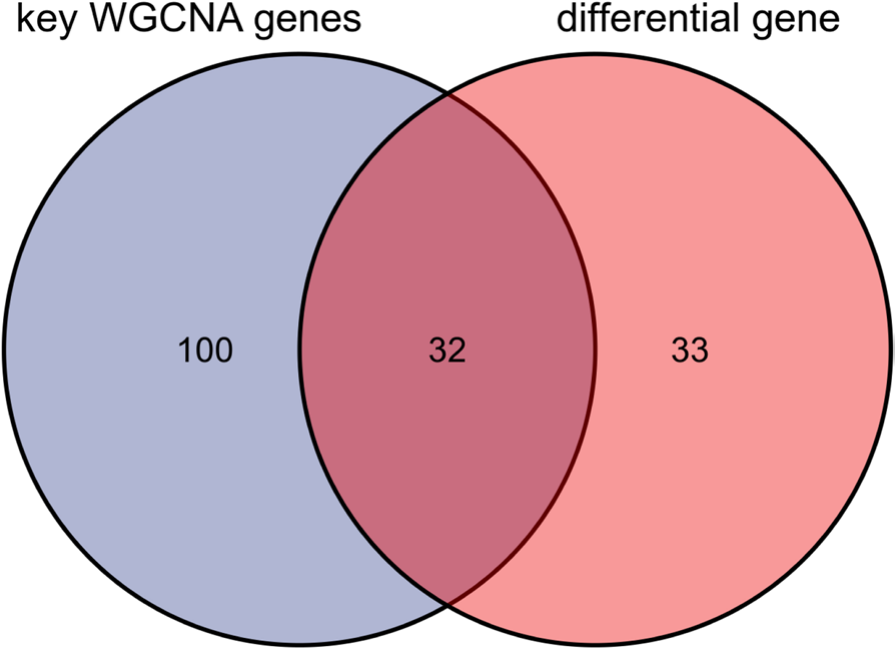
Venn diagram

**Fig. 4:**
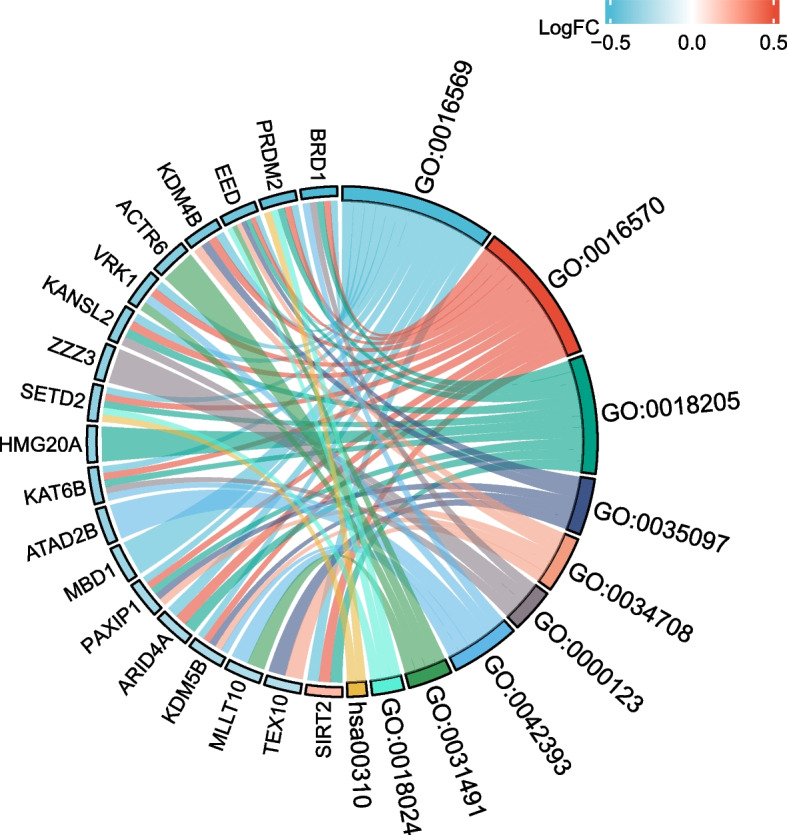
Chordal diagram for candidate key gene enrichment analysis. GO:0016569 stands for covalence chromatin modification, GO:0016570 stands for histone modification, GO:0018205 stands for peptidyl-lysine modification, GO:0035097 stands for histone methyltransferase complex, GO:0034708 stands for methyltransferase complex, GO:0000123 stands for histone acetyltransferase complex, GO:0042393 stands for histone binding, GO:0031491 stands for nucleosome binding, GO:0018024 stands for histone-lysine N-methyltransferase activity, and hsa00310 stands for lysine degradation

According to the outcomes of DEGs and the results of WGCNA core module genes, we plotted the venn diagram (Fig. 3) and identified the overlapping 32 genes as the candidate key genes in OA-related CRs (Table. S4). Then, the enrichment analysis of these 32 candidate key genes was performed through the DAVID website. The GO biological process (BP) enrichment analysis produced 132 enrichment classifications, like covalence chromatin modification, histone modification, peptidyl-lysine modification, histone methyltransferase complex, methyltransferase complex, histone acetyltransferase complex, histone binding, nucleosome binding, histone-lysine N-methyltransferase activity and other BPs. The KEGG pathway enrichment analysis produced 5 enrichment pathways, like the lysine degradation-related signal paths, cell cycle-related signal paths, nicotinate and nicotinamide metabolism-related signal paths, antigen processing and presentation-related signal paths, and TGF-β signal path (Fig. 4, and Table. S5).

### Hub gene screening results

**Fig. 5:**
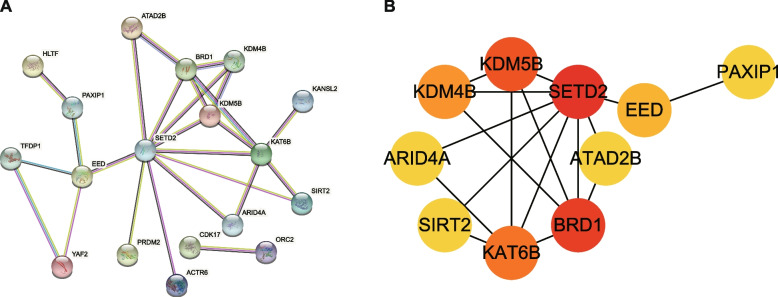
(**A**) PPI network diagram. **B** The chart of Hub Gene network interaction

Based on the aforesaid 32 key candidate genes, the String database and Cytoscape3.7.2 program were utilized to select the Hub genes in OA-related CRs. First, the key candidate genes were imported into the String database, with the confidence score > 0.4. The analysis results were exported, imported into Cytoscape 3.7.2 to establish PPI analysis maps (Fig. 5). The key protein module was screened using the cytoHubba plugin, and the top 10 CRs with Score values were selected as Hub genes, namely *SETD2* (Score = 21), *BRD1* (Score = 14), *KDM5B* (Score = 12), *KAT6B* (Score = 11), *KDM4B* (Score = 6), *EED* (Score = 4), *PAXIP1* (Score = 2), *SIRT2* (Score = 2), *ARID4A* (Score = 2), *ATAD2B* (Score = 2).

### Degree of immune cell and functional infiltration

**Fig. 6:**
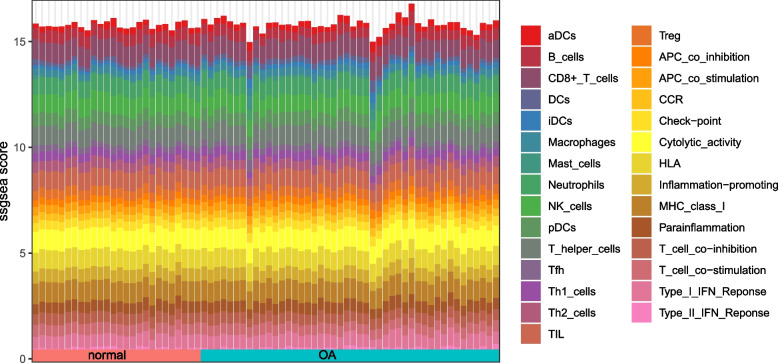
ssGSEA score profile of immune infiltration patterns in osteoarthritis samples and normal samples

The immunity degree of 29 immunocytes and functions was analyzed in the training set GSE63359 using ssGSEA (Fig. 6). The immune cell-related gene sets obtained were activated dendritic cells (aDCs), B cells, CD8+ T cells, dendritic cells (DCs), immature dendritic cell (iDCs), macrophagus, mast cells (MCs), neutrophilic cells, natural killer cells (NK cells), plasmacytoid dendritic cells (pDCs), T helper cells, T follicular helper cells (Tfh), Th1 cells, Th2-cells, tumor infiltrating lymphocyte (TIL), regulatory T cells (Treg). The immune functions included antigen-presenting cells (APC) co-inhibition, APC costimulation, chemokine C-C-Motif receptor (CCR), check-point, cytolysis activity, human leukocyte antigen (HLA), inflammation-facilitating, major histocompatibility complex class I (MHC-class-I), parainflammation, T cell co-inhibition, T cell costimulation, Type I IFN reponse, Type II IFN Reponse.

### Immune infiltration correlation and differential analysis

**Fig. 7:**
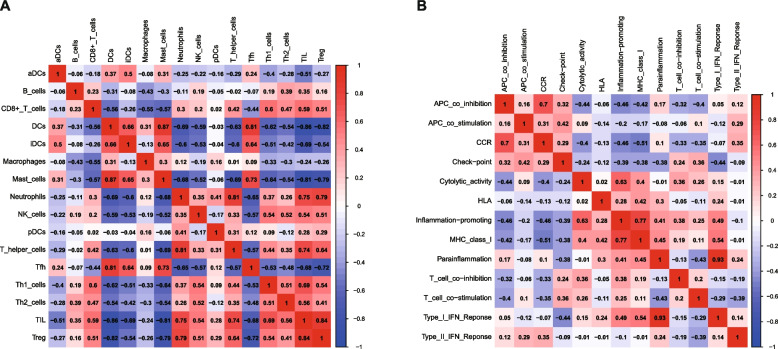
Outcomes of immunocyte infiltration correlation and difference analysis. **A** Heatmap of correlative analysis between immune cells. **B** Heatmap of correlative analysis between immune functions. In Fig. A and Fig. B, red and positive values denote positive associations, while blue and negative values denote negative associations, with darker red, darker blue, and larger absolute values representing more significant associations

The immune cell correlation heat map (Fig. 7A) demonstrates an extremely potent positive relationship between DCs and MCs (*r* = 0.87), an extremely potent positive relationship between TIL and Treg (*r* = 0.84), an extremely potent positive relationship between neutrophils and T helper cells (*r* = 0.81), an extremely potent positive relationship between DCs and Tfh (*r* = 0.81), a potent positive relationship between neutrophils and Treg (*r* = 0.79), a potent positive relationship between neutrophils and TIL (*r* = 0.75), a potent positive relationship between T helper cells and TIL (*r* = 0.74), a potent positive relationship between MCs and Tfh (*r* = 0.73). Mast cells and TIL showed an extremely potent negative relationship (*r* = − 0.81); MCs and Treg displayed a potent negative relationship (*r* = − 0.79); Tfh and Treg displayed a potent negative relationship (*r* = − 0.72). The heat map of immune function correlation (Fig. 7B) demonstrated an extremely potent positive relationship between parainflammation and Type I IFN reponse (*r* = 0.93), a potent positive relationship between inflammation-promoting and MHC-class-I (*r* = 0.77), and a potent positive relationship between APC co-inhibition and CCR (*r* = 0.7).

**Fig. 8:**
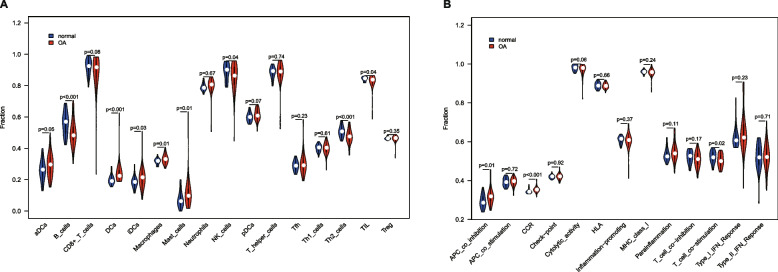
Outcomes of immunocyte infiltration differential analysis. **A** Violin plot of ANOVA between immunocytes. **B** Violin plot of ANOVA between immune functions. The blue and red areas of the graph indicate the distribution of sample density per immune cell and function, while the white dots indicate the medians

Differences in immune infiltrating cells and functions in the peripheral blood of OA patients and healthy subjects were analyzed by violin plots (Fig. 8A **and** B), with *P* < 0.05 representing significant differences. The results showed that in the OA group DCs (*P* < 0.001), iDCs (*P* = 0.03), macrophages (*P* = 0.01), MCs (*P* = 0.01), APC co-inhibition (*P* = 0.01) and CCR (*P* < 0.001) were significantly increased, whereas B cells (*P* < 0.001), NK cells (*P* = 0.04), Th2 cells (*P* < 0.001), TIL (*P* = 0.04) and T cell co-stimulation (*P* = 0.02) were significantly decreased.

### Correlation analysis between hub gene and immunocyte infiltration

**Fig. 9:**
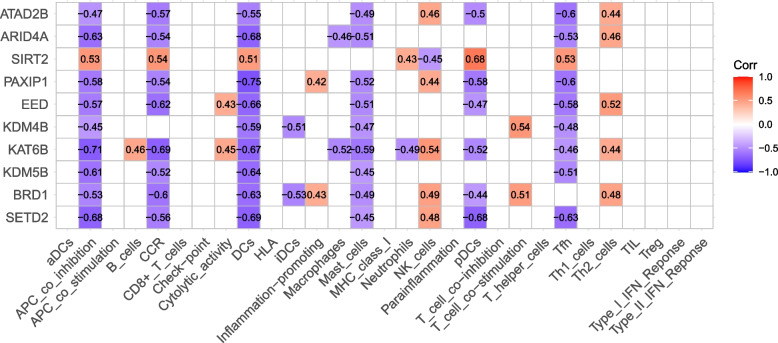
Heat map of Hub gene and immune infiltration correlation analyses. Red and positive values denote positive associations, while blue and negative values denote negative associations, with darker red, darker blue, and larger absolute values representing more significant associations

Based on the results of ssGSEA and Hub gene analyses, the association score plot of Hub genes in immunocytes and functions was obtained in this study (Fig. 9), and the outcomes revealed that the entire 10 Hub genes were significantly associated with one or more cells and functions in immune infiltration, and we selected five genes with closer association for subsequent analyses, including *BRD1*, *KAT6B*, *EED*, *PAXIP1*, and *ATAD2B*.

### Risk model construction and validation

**Fig. 10:**
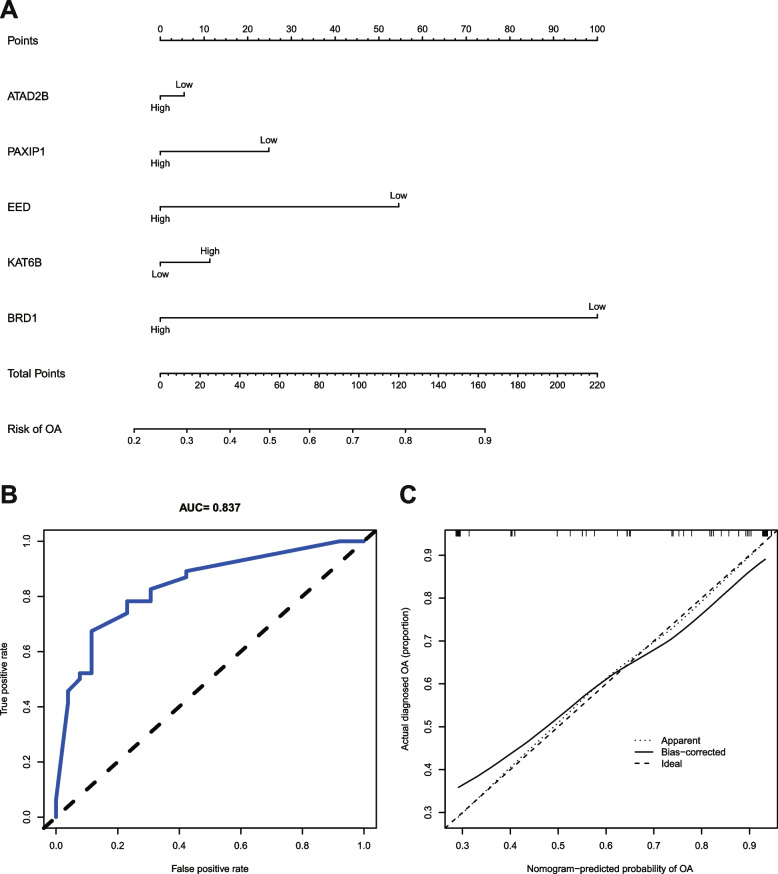
Risk model establishment and validation of the training set. **A** Nomogram plot. **B** ROC plot. **C** Calibration curve plot

**Fig. 11:**
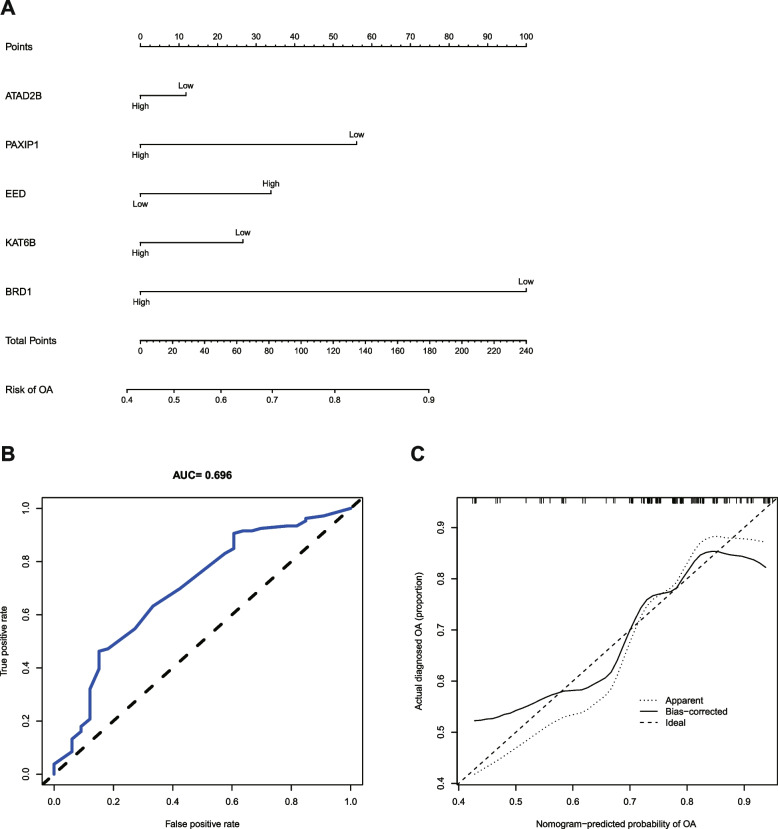
Risk model establishment and validation of the validation dataset. (**A**) Nomogram plot. (**B**) ROC plot. (**C**) Calibration curve plot

The five Hub genes (*BRD1*, *KAT6B*, *EED*, *PAXIP1*, and *ATAD2B*) from the aforesaid results were included in the risk study, and a nomograph model was established on the basis of dichotomous logistic regressive analyses to calculate the total score so as to analyze the risk probability of OA patients. The results showed that the *BRD1* (*P* = 8.97 × 10^− 3^) might be a risk factor for OA (Fig. 10A**,** Table. 1). The ROC plot (AUC = 0.837) revealed that the nomograph model exhibited a satisfactory predictive power for the assessment of risk factors in OA patients **(**Fig. 10B). The calibration curve plot showed that the nomogram model was in good agreement with the actual results (Fig. 10C). In addition, the results of multicollinearity analysis showed that the *K* value was 59.83, which was less than 100, indicating a weak multicollinearity among the five genes. The results of the validation set GSE48556 analysis were consistent with the above results, where the *P* value for *BRD1* was 4.97 × 10^− 3^, and the AUC value was 0.696 (Fig. 11**,** Table. 2).

**Table. 1:** Specifics of each risk factor (CRs) in the training dataset (GSE63359) risk model

Risk factor(CRs)	Estimate	Error	***Z***-value	***P***-value
*ATAD2B*	−0.11	0.90	−0.12	0.90
*PAXIP1*	−0.50	0.68	−0.74	0.46
*EED*	−1.11	0.79	−1.40	0.16
*KAT6B*	0.23	0.89	0.26	0.80
*BRD1*	−2.03	0.78	−2.61	8.97 × 10^−3^
Intercept	2.63	0.65	4.06	4.95 × 10^−5^

**Table. 2:** Specifics of each risk factor (CRs) in the validation dataset (GSE48556) risk model

Risk factor(CRs)	Estimate	Error	***Z***-value	***P***-value
*ATAD2B*	−0.16	0.43	−0.38	0.70
*PAXIP1*	−0.78	0.60	−1.30	0.19
*EED*	0.47	0.42	1.10	0.27
*KAT6B*	−0.37	0.58	−0.63	0.53
*BRD1*	−1.39	0.49	−2.81	4.97 × 10^−3^
Intercept	1.02	0.41	2.48	0.01

### Hub gene drug and miRNA prediction

**Fig. 12:**
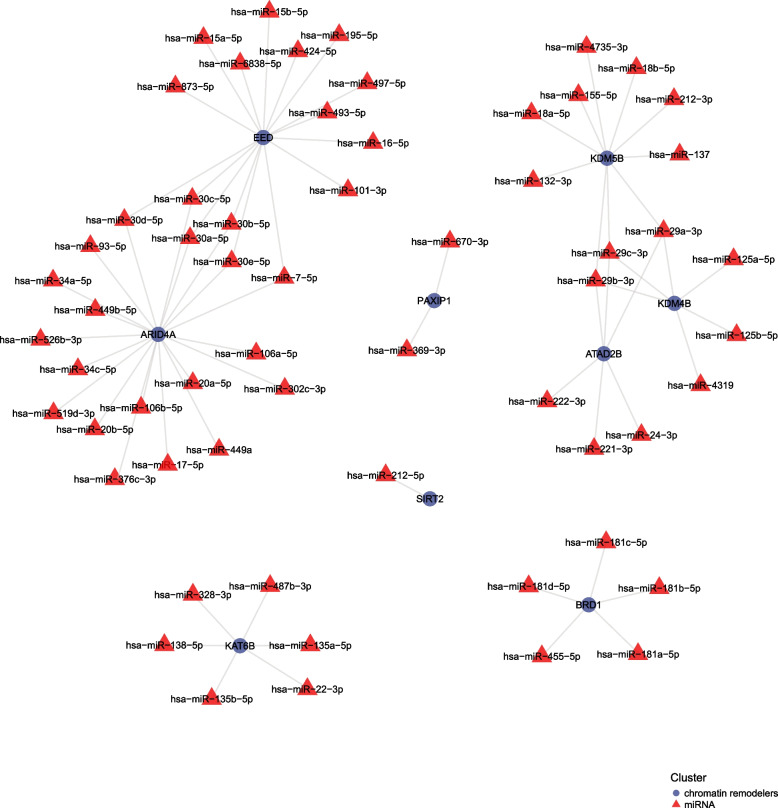
Regulatory network of Hub genes

miRNAs play an important role in modulating the expressing levels and biofunctions of protein-encoding genes. Therefore, in this study, the FunRich software was utilized to forecast the upstream miRNAs of Hub genes and we obtained a total of 60 miRNAs, such as hsa-miR-15a-5p, hsa-miR-22-3p, hsa-miR-24-3p, hsa-miR-181a-5p, hsa-miR-369-3p and so on. In addition, the interactions between the two were input into xiantao academic platform, which thus realized the visualization of the miRNA-mRNA regulatory network (Fig. 12).

## Discussion

OA is a complex and diverse disease affecting the functioning of joints in all tissues of the body. Among these, immune infiltration has been shown to play an important role in the progression of OA. For example, Tigao et al [22] reported that driving M1 macrophage polarization promoted the development of mast chondrocytes. Obesity is a risk factor for the development of OA in weight-bearing joints in humans [23]. Recent studies suggest that dysregulation of the immune system in obese patients may exacerbate the inflammatory response to OA [24]. Immune cells also play a regulatory role in the development of OA-related symptoms [25]. Nees et al. [25] found that a lower percentage of Treg in the synovial membrane was associated with increased pain and functional impairment in knee OA. In addition, recently, studies on the relevance of epigenetics in the field of OA have been conducted. Mounting proofs have revealed that epigenetic mechanisms are vital for regulating mRNA expression and local transcriptional activity in chondrocytes [26, 27]. However, there are few studies on the epigenetic mechanisms in OA immune infiltration. Thereby, we screened 32 candidate key genes for the CRs in OA by performing differential analysis and WGCNA analysis on the CR expression matrix. Then, the ten Hub genes (*SETD2*, *BRD1*, *KDM5B*, *KAT6B*, *KDM4B*, *EED*, *PAXIP1*, *SIRT2*, *ARID4A*, *ATAD2B*) were further screened by PPI analysis. Using these ten Hub genes, we performed immune infiltration correlation analysis and found that they all exhibited significant correlations with one or more cells and functions in immune infiltration. In addition, five of these Hub genes (*BRD1*, *KAT6B*, *EED*, *PAXIP1*, *ATAD2B*) were selected, which were more strongly correlated with immune infiltration, as risk factors. Dichotomous logistic regression analysis was utilized to establish a risk model, and we surprisingly discovered that *BRD1* might be an independent risk factor for OA (training dataset: *P* = 8.97 × 10^− 3^, validation dataset: *P* = 4.97 × 10^− 3^).

*BRD1*, a member of the *BRD* family, contains an N-terminal tandem bromodomains that recognizes and binds acetylated lysine residues on histones [28]. *BRD1* has been identified to be associated with the acetylation of histone 3 lysine 14 (*H3K14ac*), and this histone marking occurs at the active promoters [29]. Related studies have shown that in different cell types, inflammatory stimuli induce the occurrence of *H3K14ac* marking in the promoter regions of IL6, IL8, and TNF-α [30, 31]. *BRD1*-mediated histone acetylation is also essential for the early development of T cells [32]. *BRD1* is localized to the enhancer of CD8 gene and activates CD8 gene expression through acetylation of H3K14, thereby promoting T cell development [32]. In addition to T cells, Klein et al. [33] Observed that silencing *BRD1* reduced LPS-induced *TNF-α* expression in monocyte-derived macrophages. A recent study showed that normal mitochondrial function is dependent on the balanced expression of *BRD1*, and the loss of *BRD1* affects the downstream epigenetic effects of peroxisome proliferator-activated receptor (PPAR) expression, leading to mitochondrial dysfunction [34]. PPAR has been shown in previous studies to be required for the maturation of alternative activated macrophages. Its disruption leads to the development of diet-induced obesity in the organism [35]. In addition, mitochondria also play an important role in post-traumatic OA involving mechanobiological factors and in maintaining chondrocyte homeostasis [36, 37]. This suggests that mitochondrial modulators targeting *BRD1* will likely be an effective potential therapeutic modality for future OA treatment, especially in the immune context. The above studies and the results of our analysis exemplify the potential research value of *BRD1*-mediated inflammation and the immune system in the treatment of OA.

In addition to *BRD1*, we obtained several other Hub genes that are closely related to immunological aspects of OA. *KAT6B* gene, located in the 10q22.2 region [38], is a member of the *MYST* family of histone acetyltransferases. It can acetylate the lysine 23 residue of histone H3 (*H3K23ac*) [39]. A previous study showed that in RAW264.7 cells and macrophages, LPS stimulation resulted in the upregulation of *KAT6B* expression and *KAT6B* promoted *IL-6* production by facilitating the recruitment of *H3K23ac* to the promoter region of the *IL-6* gene, and it further revealed that variations in the expression levels of the *KAT6B* gene didn’t affect the activities of *MAPK* and *NF-κB* p65 [40]. *EED* is one of the core subunit components of the polycomb repression complex 2 (*PRC2*), which can catalyze the trimethylation of histone H3 lysine K27 (*H3K27me3*) [41]. In this complex, *EED* mainly plays the role of “reader”, i.e., binding to the methyl group, while the other complex component, *EZH1/2*, plays the role of “writer”, i.e., catalyst [42]. The epigenetic mechanisms mediated by this complex have been studied in the context of OA and immunity. For example, Dobenecker et al [43] demonstrated experimentally that the inhibition of *PRC2* greatly attenuated autoimmune diseases caused by regulatory T cell deficiency. Liu et al. found that *EZH2*-mediated epigenetic modifications inhibited *ATOH8* expression and thus promoted chondrogenesis differentiative activity of human umbilical cord derived mesenchymal stem cells (HUMSC). However, there are few studies on the roles of *EED* in osteoarthritis and immunity, hence exploring the epigenetic mechanisms mediated by *EED* in OA immune infiltration may be a novel strategy to improve the immune microenvironment of OA. *PAXIP1*, also called *PTIP*, is vital for the breakage of DNA double strand, because PAXIP1 and *53BP1* facilitate non-homologous end-joining restoration [44]. Moreover, it’s a pivotal protein in the Mll3/4 histone H3K4 methyltransferase complexes [45]. Previously, researches have revealed that *PTIP* is critical for the developmental process of B and T cells and it’s a licensing factor for the maturation of lymphocytes [46, 47]. In the research finished by Wang et al [48], *PTIP* is capable of fine-tuning the inflammation reaction of macrophagus via regulating the metabolic process of NAD+ and the expressing level of CD38 in inflammation-related macrophagus in a way that changes the acetylation state of the cis-modulatory region.

Combining the outcomes of differential immunocyte infiltration analyses and the correlations of immunocyte infiltration by CRs, it is not difficult to find that DCs, mast cells, APC co-inhibition, and CCR might be vital for the regulation of OA immune microenvironment by CRs. DCs are a group of antigen-presenting cells secreting immunity mediating factors, cell factors and chemotactic factors related to persistent inflammation illnesses [49]. A recent study showed that the demethylation inhibitor enzyme *GSK-J4* enhanced epigenetic signaling activation and reduced suppressive marks in the promotors of retinaldehyde dehydrogenase isoforms 1 and 3 in DCs, thereby attenuating inflammation colitis via decreasing the inflammation potential of DCs [50]. MCs are rapid responders of the natural immune system and can respond rapidly to exogenous pathogens and endogenous danger signals [51]. A recently finished clinical research revealed that the mean number of MCs, synovitis score and quantity of vessels were remarkably greater in the OA subjects versus the controls [52]. In addition, the study of Folkerts et al [53] revealed that short-chain fatty acids reduced the acetylation of promoter regions such as BTK, SYK, and LAT in human mast cells, thereby inhibiting mast cell-mediated inflammatory diseases to some extent. APCs are a type of helper cells in the body with the ability to take up, process and deliver antigenic information, which can induce immune responses from T and B cells. A previous experiment showed that menstrual blood-derived mesenchymal stem cells could reduce the inflammatory response triggered by transplantation by inhibiting antigen-presenting cells [54]. CCR2 is one of the more studied chemokine receptors in OA. CCR2 is upregulated in interleukin-17A-treated chondrocytes and protects type II collagen synthesis and attenuates the IL-17A-induced overexpression of *RUNT*-related transcription factor 2 by inhibiting CCR2 expression, thereby delaying the development of chondrocyte hypertrophy [55].

In addition, the current study also predicted the upstream miRNAs of OA-related CRs by the FunRich software, with a total of 60 miRNAs obtained. As the quantity of bio-sequencing data elevates, forecasting the relationship between ncRNAs and illnesses via bioinformatic analyses is highly beneficial for subsequent biological experiments [56]. Predicting and quantifying the relationship between human ncRNAs and diseases through big data analyses can validly discover the most correlated RNA-diseases for experiment-based validation, thus uplifting the efficiency and reducing the expense of bioexperiments [57]. However, there are still certain limitations in this study: First, although the microarray data used met the sample size requirement, there remains a possibility that the sample size is insufficient, which may lead to certain bias in the final results. Second, although OA-related CRs were identified, our team failed to elucidate their specific mechanisms of actions, which warrants more researches in the future. However, in general, the results acquired herein harbor certain reference value for subsequent basic studies on the epigenetics in OA immunity, and may narrow the screening scope to some extent.

## Supplementary Information

**Additional file 1:** **Table S1.** The chromatin regulators provided by the study of Lu et al. (Table. S1 chromatin regulators.xlsx)

**Additional file 2:** **Table S2.** Differential expression specifics of 65 DEGs.

**Additional file 3:** **Table S3.** Gene profile of each module in WGCNA analysis.

**Additional file 4:** **Table S4.** Overlapping genes between DEGs and WGCNA core module genes.

**Additional file 5: Table S5.** Gene ontology and KEGG pathway enrichment analysis of candidate key genes in OA-associated CRs.

## Data Availability

The GSE63359, GSE51588, and GSE117999 datasets used in this study can be found in the Gene Expression Omnibus (https://www.ncbi.nlm.nih.gov/geo/).

## Abbreviations

CRs: Chromatin regulators
OA: Osteoarthritis
WGCNA: Weighted gene co-expression network analysis
PPI: Protein-protein interaction
*LSD1*: Lysine-specific demethylase-1
GEO: Gene Expression Omnibus
FC: Fold change
TOM: Topological overlap matrix
MsigDB: Molecular Signatures Database
ssGSEA: Single-sample gene set enrichment analysis
ROC: Receiver operating characteristic
DEGs: Differentially expressed genes
BP: Biological process
DCs: Dendritic cells
iDCs: Immature dendritic cell
MCs: Macrophagus, mast cells
NK cells: Natural killer cells
pDCs: Plasmacytoid dendritic cells
Tfh: T follicular helper cells
TIL: Tumor infiltrating lymphocyte
Treg: Regulatory T cells
APC: Antigen-presenting cells (APC)
CCR: Chemokine C-C-Motif receptor
HLA: Human leukocyte antigen
MHC-class-I: Major histocompatibility complex class I
*H3K14ac*: Histone 3 lysine 14
PPAR: Proliferator-activated receptor
*PRC2*: Polycomb repression complex 2
